# Mitochondrial Haplotype Diversity in Zambian Lions: Bridging a Gap in the Biogeography of an Iconic Species

**DOI:** 10.1371/journal.pone.0143827

**Published:** 2015-12-16

**Authors:** Caitlin J. Curry, Paula A. White, James N. Derr

**Affiliations:** 1 Interdisciplinary Program of Genetics, Department of Veterinary Pathobiology, College of Veterinary Medicine and Biomedical Sciences, Texas A&M University, College Station, TX, United States of America; 2 Center for Tropical Research, Institute of the Environment and Sustainability, University of California Los Angeles, Los Angeles, CA, United States of America; University of Innsbruck, AUSTRIA

## Abstract

Analysis of DNA sequence diversity at the 12S to 16S mitochondrial genes of 165 African lions (*Panthera leo*) from five main areas in Zambia has uncovered haplotypes which link Southern Africa with East Africa. Phylogenetic analysis suggests Zambia may serve as a bridge connecting the lion populations in southern Africa to eastern Africa, supporting earlier hypotheses that eastern-southern Africa may represent the evolutionary cradle for the species. Overall gene diversity throughout the Zambian lion population was 0.7319 +/- 0.0174 with eight haplotypes found; three haplotypes previously described and the remaining five novel. The addition of these five novel haplotypes, so far only found within Zambia, nearly doubles the number of haplotypes previously reported for any given geographic location of wild lions. However, based on an AMOVA analysis of these haplotypes, there is little to no matrilineal gene flow (Fst = 0.47) when the eastern and western regions of Zambia are considered as two regional sub-populations. Crossover haplotypes (H9, H11, and Z1) appear in both populations as rare in one but common in the other. This pattern is a possible result of the lion mating system in which predominately males disperse, as all individuals with crossover haplotypes were male. The determination and characterization of lion sub-populations, such as done in this study for Zambia, represent a higher-resolution of knowledge regarding both the genetic health and connectivity of lion populations, which can serve to inform conservation and management of this iconic species.

## Introduction

In Zambia, the African lion (*Panthera leo*) is broadly but irregularly distributed across approximately 167,000 km^2^ of managed habitats comprised of national parks (NP) and game management areas (GMAs). Recent estimates propose the total number of wild lions in Zambia to be between 1000 to 2000 individuals [1–4]. The largest numbers are reported from the Luangwa Valley ecosystem located in the eastern part of the country (density of 2.0 [5] to 4.0 [1] lions per 100 km^2^), with the second largest concentration of lions located in the Kafue ecosystem in the west (density of 1.5 [5] to 1.83 [6] lions per 100 km^2^). Only recently have Zambia’s lions come under more intensive scientific investigation [1, 5–8]. A study utilizing nuclear microsatellite markers and the Cytochrome-b mitochondrial marker established Zambian lions in a larger-scale genetic perspective [8] and showed that Zambian lions exhibited some intermixing of genetic profiles found in eastern and southern Africa. Despite these intriguing findings and the importance of the geographic location of this range state in relation to other countries where lions occur, there remains little information regarding genetic diversity or population sub-structure of lions within Zambia.

**Fig 1:**
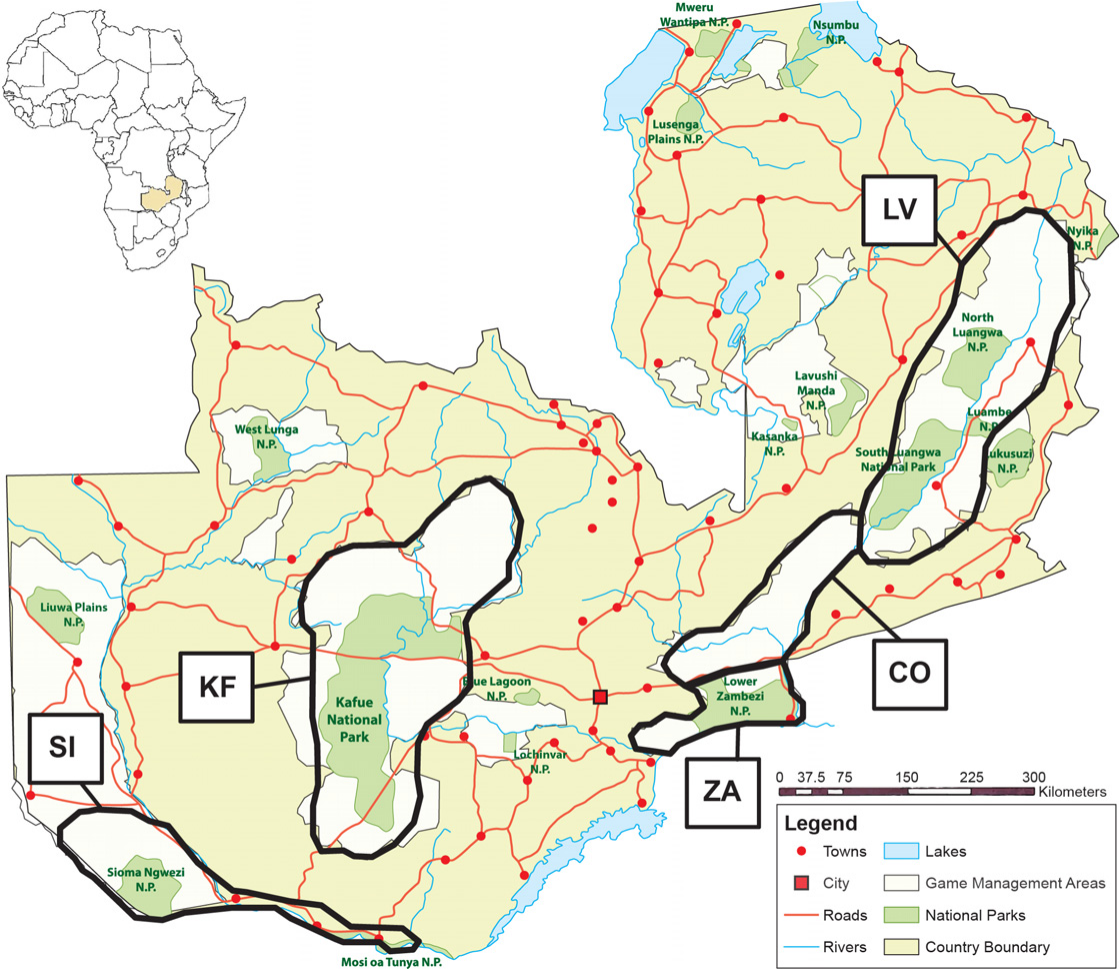
Map of Zambia showing the five main areas sampled: LV (Luangwa Valley); CO (Corridor); ZA (Lower Zambezi); KF (Kafue); and SI (Sioma Ngwezi). Eastern region consists of LV, CO and ZA. Western region consists of KF and SI. More detailed location information for each sample is available in S1 Table.

For this study, we calculated the extent of genetic diversity and matrilineal distribution in Zambian lion populations through the analysis of the 12S to 16S mitochondrial genes (mtDNA) of 165 lions found in five main areas in Zambia (Fig 1). Through extensive sampling of individuals from NPs as well as GMAs, we achieve a finer resolution image of population genetics of lions found throughout Zambia.

Mitochondrial DNA (mtDNA) is maternally inherited and has a relatively fast mutation rate that results in significant variation in mtDNA sequences. Lion prides typically consist of 2–18 related females born to that pride with 1–7 males who migrate into the pride from elsewhere [9]. When male dispersal is high while female dispersal is low, as is true in African lions, it is possible to detect geographic structure through the use of mtDNA [10].

The 12S and 16S genes of the mtDNA encode ribosomal RNAs (rRNA) necessary for the translation of messenger RNAs into mitochondrial proteins. The 12S and 16S genes are more conserved than the protein-coding genes of mtDNA [11] with 12S slightly more conserved than 16S [12]. Due to this conservation, haplotype diversity represents a deeper, more historic level of diversity within the population. Levels of genetic diversity are directly proportional to a species’ ability to adapt, survive and thrive. Therefore, loss of genetic diversity is detrimental to overall population health and long-term survival because it decreases the population’s potential to adjust to environmental changes or perturbations. We consider our findings at the population and sub-population scale and discuss potential ramifications of genetic sub-structure for lion management and conservation.

## Methods

### Sample Collection

African lion DNA samples (hair, skin, bone and/or tissue) were collected during research conducted by the Zambia Lion Project (ZLP) while operating in partnership with the Zambia Wildlife Authority (Research/Employment Permit No. #008872). Samples were collected between the years of 2004–2012 from dried skins of trophy hunted lions, biopsy darting of free-ranging live lions, and tissue or skin samples of “problem” lions killed by the Zambia Wildlife Authority. The Zambia Wildlife Authority includes a research division and veterinary division that reviews all proposed studies, including animal care and use protocols, and approves studies only after they have met department standards. For this study, in addition to the research division’s review of the proposal, Zambia Wildlife Authority’s chief veterinarian reviewed the sampling protocol and examined the veterinary projector, cartridges, and biopsy darts prior to approval. Review of the proposal included interviews with ZLP’s Principal Investigator, P.A. White to discuss in detail the sampling protocol and field testing of the biopsy darting equipment.

Lion skin was obtained by collecting a small (1x1cm) snip of dried skin with hair attached and storing it in individually labeled paper envelopes. Trophy lion skins were from male animals previously sport hunted under strict permitting by Zambia Wildlife Authority and in accordance with national hunting regulations. Problem lion skins were male and female animals destroyed by Zambia Wildlife Authority. No lions were sacrificed specifically for Zambia Lion Project. Where skins were not available to sample, small (1x2cm) fragments of turbinate bones were collected from the nasal passages of cleaned skulls.

Biopsy tissue samples were collected from live lions using a 4x scoped Pneu-dart Model 389 cartridge fired veterinary projector that propelled a 3cc Pneu-dart biopsy dart specially designed for use on African lion (Pneu-dart, Williamsport, PA). Darts were fired from a range of 15–60m using green CCI power loads. Prior to firing a dart, a rangefinder was used to gauge distance to the lion. A 5-position pressure control dial on the projector allowed the power of the dart to be safely controlled over a broad range of darting distances. The tip of the biopsy dart contained a cutting farrel that upon impact punched a plug 3mm in diameter x 5mm in length from the lion’s shoulder or rump. The dart, which contained no drugs, bounced off immediately following impact retaining the tissue plug on a barb inside the farrel. Both male and female lions older than approximately one year of age were biopsy darted. Cubs younger than one year old were not sampled.

Tissue samples were immediately removed from the dart using sterile tweezers and placed into individually labeled vials containing 95% EtOH. Skin and tissue samples were stored in Zambia at room temperature until being transferred to a USA laboratory for analysis. Samples were collected and imported in full compliance with specific legal national and international permitting requirements. Samples were imported to the USA under CITES permits numbers #25393 and #30208.

DNA samples were obtained from both male and female lions throughout Zambia’s National Parks and GMAs making this dataset representative of Zambia’s countrywide lion population. A continuous sequence of the 12S-16S genes (1880–1882 base pairs) was analyzed from sequences successfully amplified from 165 lions (119 males, 45 females, 1 unknown; Table 1) found in five main areas in Zambia (Fig 1). These areas include five national parks (North Luangwa, South, Luangwa, Lower Zambezi, Kafue and Sioma Ngwezi) and twenty-nine GMAs.

**Table 1:** Number of males (♂), females (♀), and with unknown gender (?) for each haplotype is indicated for all areas sampled in Zambia along with the haplotype frequencies. Haplotypes H1, H9 and H11 were previously described by Antunes et al. [13]. Haplotypes Z1-Z5 are novel.

		*Eastern Region*									*Western Region*								
		LV			CO			ZA			KF			SI					
	**Haplotype**	**♀**	**♂**	**?**	**♀**	**♂**	**?**	**♀**	**♂**	**?**	**♀**	**♂**	**?**	**♀**	**♂**	**?**	n	**ƒ**	**s.d.**
H1											0	1	0				1	0.0061	0.0061
H9		0	1	0							18	41	0	0	1	0	61	0.3697	0.0377
H11		6	13	0	3	5	0	1	0	0	0	1	0				29	0.1758	0.0297
Z1		7	32	1	0	6	0	1	0	0	0	4	0				51	0.3091	0.0361
Z2								0	1	0							1	0.0061	0.0061
Z3											5	10	0				15	0.0909	0.0224
Z4											0	2	0				2	0.0121	0.0085
Z5											4	1	0				5	0.0303	0.0134
	n	13	46	1	3	11	0	2	1	0	27	60	0	0	1	0	165		
		60			14			3			87			1					
	A	3			2			3			7			1			8		

n = sample size; for ♂, ♀ and? for each area and by area, haplotype and total.

A = number of haplotypes.

ƒ = frequency.

s.d. = standard deviation.

### Molecular Analysis

To allow for a direct comparison with previously published data, we used the same maternal sequence (mtDNA) assessed by Antunes et al. [13] whose analysis did not include this region of Africa. The *Panthera* genus has a large 12.5 kb integration of mtDNA into the nuclear genome, or numt, [14] which could be a potential source of error during analysis. False sequences of mtDNA/numt recombinants produced during PCR [15] can result in inaccurate levels of genetic diversity. To prevent potential numt amplification, mtDNA specific primers for the 12S-16S region designed by Antunes et al. [13] to prevent numt amplification were used.

DNA isolation, PCR and DNA sequencing and analysis were completed using standard laboratory techniques in the DNA Technologies Core Laboratory at Texas A&M University in College Station, TX (http://vetmed.tamu.edu/dnacore; details in S1 Appendix). PCR amplification was conducted using the KAPA Biosystems KAPA2G™ Robust HotStart PCR Kit according to manufacturer’s instructions. The cycling profile was as follows: initial denaturation at 95°C for 3 min, then denaturation, primer annealing and extension at 95°C for 15 s, 55°C for 15 s and 72°C for 45 s for 35 cycles, followed by a 1 min extension at 72°C. Samples were then cooled and held at 4°C until sequencing. PCR products were sequenced on an Applied Biosystems 3130xl Genetic Analyzer then aligned, manually edited and assigned a haplotype using SEQUENCHER v4.8 [16].

### Statistical Analysis

Genetic diversity calculations were implemented using Arlequin v3.5 [17]. The number of polymorphic sites, gene diversity, nucleotide diversity and haplotype frequency estimations were calculated as a single population. Lions were divided into sub-populations and combined regionally for intra-population calculations of the coefficient of differentiation (Fst) and hierarchical analyses of molecular variance (AMOVA). Pairwise differences (π) between and within populations were computed along with Nei’s distance (d) through the use of conventional F-statistics.

Phylogenetic analysis included all haplotypes from Antunes et al. [13] (GENBANK Accession #s FJ151641-FJ151652) and novel haplotypes found in this study (GENBANK Accession #s KT164799-KT164803). The tree was rooted by the tiger (*Panthera tigris*) with a sequence from the complete mitogenome (GENBANK Accession #KJ508413) which was aligned to the lion sequences then trimmed to contain the same regions. Phylogenetic analysis was performed using ML and Bayesian inference methods. ML analysis was then performed using Garli v2.01 [18], RAxML [19], and PhyML [20]. A Bayesian analysis was conducted in Mr. Bayes [21–22] via Markov chain Monte Carlo (MCMC). Samples were drawn every 1,000 steps over 50,000,000 MCMC steps. The first 10% were discarded as burn-in. Acceptable sampling and convergence to the stationary distribution were checked by inspection of traces using Tracer v1.5 [23] and trees were visualized using FigTree v1.4.0 [24]. In addition, a haplotype network was formed utilizing the median-joining option of Network v4.6.1.3 [25].

## Results

When considered as one population, gene diversity throughout the population of lions in Zambia was high at 0.7319 +/- 0.0174. AMOVA analysis, run with each of the main areas within Zambia grouped regionally as an eastern [Luangwa Valley (LV), Corridor (CO) and Lower Zambezi (ZA)] and a western [Kafue (KF) and Sioma Ngwezi (SI)] sub-population (Fig 1), resulted in an Fst of 0.47 (p-value<0.001) between regional sub-populations (Table 2). Within the eastern sub-population, Fst calculated between areas was 0.05. Fst was not calculated between areas within the western sub-population due to SI contributing only one sample that would have skewed the result. Gene diversity was equal but decreased slightly when the population was separated regionally (eastern at 0.5057 +/- 0.0575, western at 0.5014 +/- 0.0336).

**Table 2:** AMOVA results with Fst. Percent variation is given among populations (Va) and within groups (Vb). The significance of differentiation within and among populations was tested by 1,000 permutations.

Source of Variation	d.f.	Sum of Squares	Variance Components	Percentage of Variation	p-value
Among Populations	1	18.966	0.22785 Va	47.50	<0.001
Within Populations	163	41.052	0.25185 Vb	52.50	<0.001
Total	164	60.018	0.47971		
**Fixation Index**	*Fst*:	0.47499			

**Fig 2:**
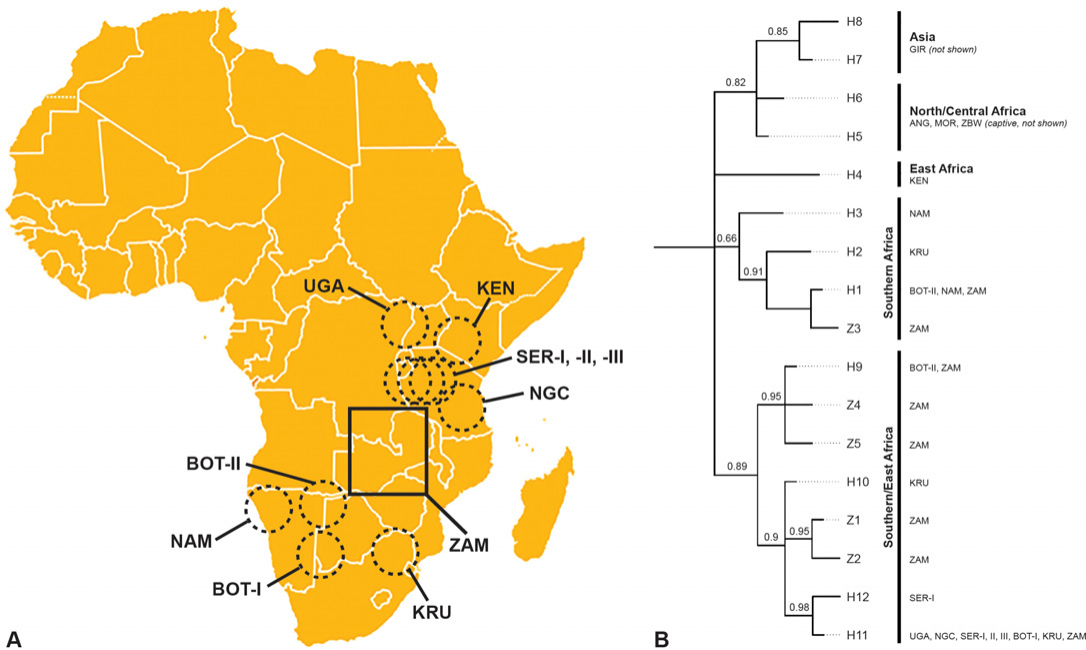
Geographic location of lion samples and phylogenetic relationship of 12S-16S. (A) Range-wide map of lions sampled. Circles indicate geographic locations for populations determined by Antunes et al. [13]. Zambia is denoted by a square. All locations aside from ZAM (Zambia) were established by Antunes et al. [13]: UGA (Uganda); KEN (Kenya); SER (Serengeti National Park, Tanzania); NGC (Ngorongoro Crater, Tanzania); KRU (Kruger National Park, South Africa); BOT-I (Southern Botswana and Kalahari, South Africa); BOT-II (Northern Botswana); NAM (Namibia); GIR (Gir Forest, India); ANG (Angola); ZBW (Zimbabwe); and MOR (Morocco). (B) Bayesian analysis with posterior probability values on the nodes. H1-H12 are haplotypes that were described by Antunes et al. [13] and Z1-Z5 are novel haplotypes so far only found within Zambia.

Eight haplotypes were found; three haplotypes (H1, H9, H11) described by Antunes et al. [13] and five previously unreported haplotypes (Z1, Z2, Z3, Z4, Z5). The previously unreported haplotypes were regarded as true, novel haplotypes when they appeared two or more times. Haplotypes that appeared only once were verified through re-sequencing before being regarded as true, novel haplotypes. Of the five novel haplotypes, three were considered rare with frequencies below 5% (Table 1). Of the three previously described haplotypes, H1 and H9 were found in northern Botswana and Namibia while H11 was found throughout eastern Africa spanning from Uganda across the Serengeti to the Ngorongoro Crater in Tanzania as well as in southern Botswana and Kruger National Park in South Africa (Fig 2).

Z1, found countrywide, differs from H10 by only one base pair. H10 is a haplotype seen only in Kruger National Park and is found with H2 (also seen only in Kruger National Park) and H11 (2 base pair differences from Z1, also countrywide). Haplotype Z2, which only appeared once, was verified through re-sequencing. Haplotype Z2 differs from haplotype Z1 by only one polymorphic site. This polymorphic site is also the only transversion, an adenine (purine) to a thymine (pyrimidine) substitution, seen at any polymorphic site between all haplotypes (as shown in Table 3, *position 1801*). The nucleotide differences by position and the number of base pair differences among haplotypes are shown in Table 3 and S2 Table, respectively. Z3 has two insertions, similar to H1. Z3 was prevalent in KF and was the fourth most common haplotype overall with a frequency of 0.091. In contrast, H1 appeared only once (frequency = 0.006) in a sample from KF but was found elsewhere in Northern Botswana and Namibia. Z4 and Z5, seen only in KF, each differ from H9, the predominant haplotype of KF (frequency = 0.370), by only one base pair each.

**Table 3:** Nucleotide position for each polymorphic site.

	*242*	*369*	*393*	*462*	*513*	*531*	*571*	*596*	*631*	*684*	*695*	*732*	*798*	*812*	*840*	*841*	*928*	*929*	*961*	*1039*	*1082*	*1140*	*1220*	*1247*	*1328*	*1387*	*1610*	*1629*	*1632*	*1646*	*1801*
**H1** *	C	C	A	T	C	G	G	A	T	T	G	C	T	G	T	A	A	A	A	C	C	A	A	T	A	T	C	C	T	C	A
H2	•	•	•	•	•	A	•	•	•	•	•	•	•	•	C	•	•	-	•	•	•	•	•	C	•	•	•	T	•	•	•
H3	•	•	•	•	T	A	•	•	•	•	•	•	•	•	•	•	•	-	G	•	•	•	G	•	•	•	•	T	•	•	•
H4	•	•	•	C	•	A	•	•	•	•	A	T	C	•	•	•	-	-	G	•	T	•	•	•	•	•	T	T	•	T	•
H5	•	T	•	•	•	A	•	•	C	•	•	•	•	•	•	•	-	-	G	•	•	•	•	•	•	•	•	T	•	T	•
H6	•	T	•	•	•	A	•	•	C	•	•	•	•	A	•	•	-	-	G	•	•	•	•	•	•	•	•	T	•	T	•
H7	T	T	•	•	•	A	•	•	C	•	•	•	•	•	•	•	-	-	G	•	•	G	•	•	•	•	•	T	•	T	•
H8	T	T	•	•	•	A	•	•	•	C	•	•	•	•	•	•	-	-	G	•	•	G	•	•	•	•	•	T	•	T	•
**H9**	•	•	•	•	•	A	•	•	•	C	•	•	•	•	•	G	-	-	G	•	•	•	•	•	G	•	•	T	•	T	•
H10	•	•	•	•	•	A	•	•	•	C	•	•	•	•	•	•	-	-	G	•	•	•	•	•	G	C	•	T	•	T	•
**H11**	•	•	•	•	•	A	•	•	•	C	•	•	•	•	•	•	-	-	G	T	•	•	•	•	G	C	•	T	•	T	•
H12	•	•	•	•	•	A	A	•	•	C	•	•	•	•	•	•	-	-	G	T	•	•	•	•	G	C	•	T	•	T	•
**Z1**	•	•	•	•	•	A	•	•	•	C	•	•	•	•	•	•	-	-	G	•	•	•	•	•	G	C	•	T	C	T	•
**Z2**	•	•	•	•	•	A	•	•	•	C	•	•	•	•	•	•	-	-	G	•	•	•	•	•	G	C	•	T	C	T	T
**Z3**	•	•	•	•	•	•	•	•	•	•	A	•	•	•	•	•	•	•	•	•	•	•	•	•	•	•	•	•	•	•	•
**Z4**	•	•	G	•	•	A	•	•	•	C	•	•	•	•	•	G	-	-	G	•	•	•	•	•	G	•	•	T	•	T	•
**Z5**	•	•	•	•	•	A	•	G	•	C	•	•	•	•	•	G	-	-	G	•	•	•	•	•	G	•	•	T	•	T	•

*All nucleotide polymorphisms in 12S-16S are shown for haplotype H1. For all other haplotypes, only nucleotides that differ from H1 are shown.

Haplotypes found in Zambia are in **bold**.

**Fig 3:**
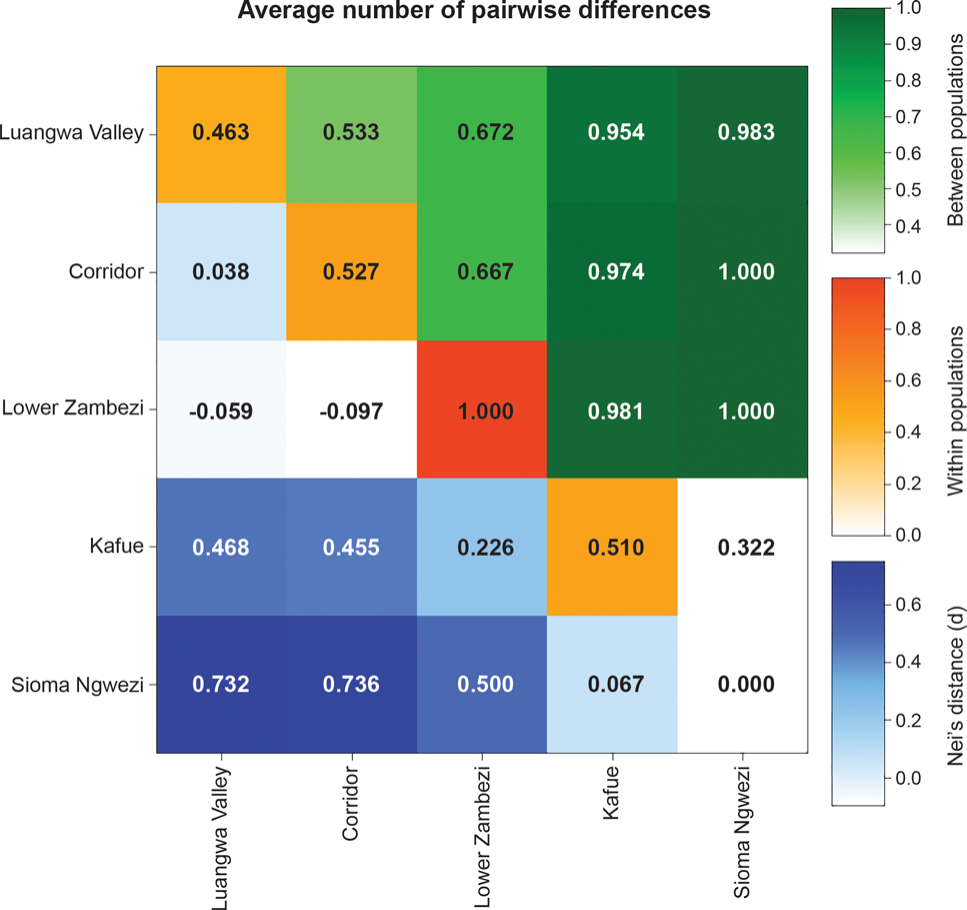
Nei’s distance (d) and average number of pairwise differences within and between populations.

Average number of pairwise differences and Nei’s distance (d) are shown in Fig 3. Nei’s distance (d) and between population pairwise differences were highest between an eastern (LV, CO or ZA) and western area (KF or SI). While the ZA shows the highest level of within population pairwise differences (1.0), this is due to all three samples from this area having different haplotypes. Areas with higher sample sizes (KF, CO, LV) exhibit similar levels of within population pairwise differences (0.51, 0.53, 0.46, respectively).

**Fig 4:**
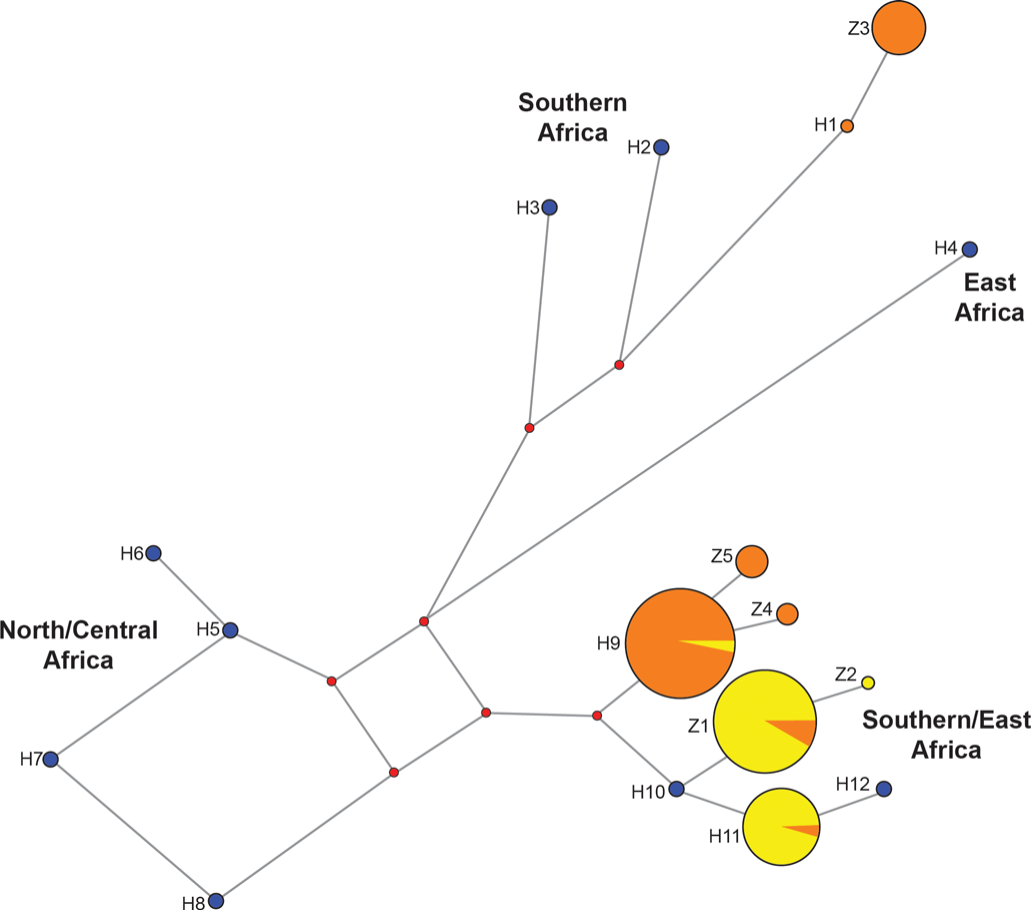
Median-joining network of 12S-16S haplotypes. Orange indicates haplotypes found in the western sub-population and yellow indicates haplotypes found in the eastern sub-population. Circle sizes of haplotypes found in Zambia are proportional to haplotype frequency. Red circles indicate median vectors. Blue circles indicate haplotypes not found in Zambia. H1-H12 are haplotypes which were described by Antunes et al. [13] and Z1-Z5 are novel haplotypes so far only found within Zambia.

It is necessary to look at the occurrence of haplotypes range-wide and how haplotypes in neighboring areas compare to one another to understand the diversity present. A comparison of molecular diversity indices and nucleotide composition for haplotypes found within Zambia versus all haplotypes range-wide are shown in Table 4. Phylogenetic analysis was conducted to bring the Zambian population into context with the entire range of the African lion. The Bayesian analysis is presented (Fig 2B) supported by posterior probability values of >60% for all nodes. While all trees resulted in similar clustering, Bayesian posterior probabilities offered stronger support than Maximum Likelihood (ML) bootstrap values. Four regionally grouped clusters can be identified for *Panthera leo*–Asia/Central/Northern Africa, East Africa, Southern Africa and Southern/East Africa. The Southern/East Africa group consists of two branches, one containing south central Africa (Botswana and Zambia) and the other eastern Africa from South Africa northwards to Kenya. The same regional clusters could be found in the haplotype network (Fig 4).

**Table 4:** Molecular diversity indices and nucleotide composition.

*Within*:	*Zambia*	*Range-wide*
Nucleotide Sites:	1882	1882
# of Haplotypes:	8	17
Polymorphic Sites:	16	31
Transitions:	13	28
Transversions:	1	1
Indels:	2	2
***Nucleotide Composition***		*** ***
C:	22.11%	22.07%
T:	22.67%	22.71%
A:	36.64%	36.68%
G:	18.58%	18.54%

## Discussion

Whether considered as a single population or two sub-populations, information from this study support the idea that Zambian lions represent a genetically diverse and healthy population. Gene diversity, as defined here, represents the probability that any two sampled individuals within the population will have different haplotypes [17]. The overall gene diversity for Zambian lions sampled in this study was high (0.7319+/-0.0174). Even when considered as two sub-populations, gene diversity in Zambia’s lions was higher than reported for lions in other regions (Kruger = 0.41, Namibia = 0.21, Northern Botswana = 0.29, and Serengeti = 0.03) [13]. Zambia’s eastern and western sub-populations each showed similar gene diversity (approx. 0.5). The decrease in gene diversity from a single Zambian population to two regional sub-populations is due to some haplotypes occurring in only one of the two sub-populations and the presence of crossover haplotypes, which occur in both sub-populations (H9, H11 and Z1) but are common in one and rare in the other.

While gene diversity countrywide was high, matrilineal gene flow between regional sub-populations appeared to be low. Fst between regional sub-populations was high at 0.47 (p-value<0.001) while Fst calculated within a regional sub-population was 0.05. Values of Fst greater than 0.25 suggest there is a high level of genetic differentiation between populations; a result of low gene flow [26]. Further evidence of differentiation at the sub-population scale is provided by Nei’s distance (d) measures being highest between the eastern and western regions (Fig 3). Higher distance measures assume differences are caused more by mutation and genetic drift as opposed to migration, suggesting a low number of migrants between regions [27]. When considered as two regional sub-populations, the high Fst and distance values between the eastern and western sub-populations coupled with low Fst and distance values between areas within sub-populations suggests there to be little to no matrilineal gene flow between the eastern and western sub-populations while there is considerable movement within the eastern and western sub-populations.

Phylogenetic analysis is consistent with previous studies that postulate eastern-southern Africa as being the evolutionary cradle of the lion [28–29], supporting the hypothesis that Zambia may act as a genetic corridor between lion populations in eastern and southern Africa. The haplotypes present in the Zambian lion population were also found in both the Southern Africa lineage as well as the Southern/East Africa lineage described by Antunes et al. [13]. This grouping is parsimonious with studies that examined HVR1 and Cytochrome b mtDNA sequences ([28, 29] respectively) which grouped lions into two clusters, with Zambian lions falling within the Eastern and Southern Africa cluster. Cytochrome b analysis determined the Eastern and Southern Africa cluster to be more diverse than the North, West and Central cluster although the former cluster had weaker support [8]. Dubach et al. [8] also reported a lack of gene flow between most lion conservation units (LCUs) although microsatellite analysis indicated a high level of admixture in Botswana, Namibia and Zambia.

Analysis of mtDNA data indicates minimal gene flow between Zambia’s two sub-populations; however, because it only establishes matrilineal distribution, whether the two sub-populations have historically experienced greater gene flow through higher levels of dispersal or if geographic separation has always inhibited lion movements between the eastern and western regions is unknown. Limited dispersal may still occur but not at a rate sufficient enough to maintain or increase the frequency of crossover haplotypes (i.e. H9 in LV and H11 in KF). All individuals with crossover haplotypes were male, a pattern consistent with a genetic population structure of high male dispersal and low female dispersal [10]. In African lions, males are more likely to disperse across farther distances [9, 30–32] and are, therefore, more likely to cross geographic barriers but are unable to pass on mtDNA genes.

The most widely dispersed haplotype was H11 (Fig 2). Primarily an East Africa haplotype, it was the only haplotype observed in a population in southern Botswana and is also found in low frequency in Kruger National Park [13]. The wide range of the H11 haplotype could indicate that it is an ancestral haplotype and/or that, historically, there may have been some dispersal. Lion translocations could also be a contributor to the range of this haplotype as previous microsatellite analysis has shown evidence of translocation within LCUs [8].

With translocation becoming a well‐practiced technique to prevent inbreeding within populations closed to dispersal or immigration [33], it must be determined whether there needs to be a focus on maintaining genetic diversity throughout the entire population or if there needs to be a more narrowed focus to prevent the loss of genetic diversity between sub-populations. In the example of Zambia, the question may be whether to prioritize maintaining genetic diversity throughout the country as a single population or if a more narrowed focus could serve to prevent the loss of genetic diversity between regional sub-populations. AMOVA analysis revealed little to no gene flow between the two sub-populations of lions within Zambia, a lack of genetic connectivity likely attributable to an expanse of cities and roads that inhibit modern day dispersal.

Further research including the addition of microsatellite analysis is being done to better quantify the level of overall genetic diversity within the population. The combination of mtDNA with nuclear markers will give a clearer picture to examine population-wide gene flow, identify evolutionarily distinct populations and calculate effective population size.

The findings of this study coincide with range-wide studies that propose lions are structured by region due to a lack of widespread movement of lions [8, 13, 34]. Existing regulatory measures aimed at improving lion conservation consider African lion at the species (P*anthera leo)*[35–36] or subspecies (*Panthera leo ssp*. *leo* [37]) level, with the Asiatic lion always considered as a subspecies (*Panthera leo persica* [35, 38], *Panthera leo ssp*. *persica* [36]). Alternatively, some studies have considered management of lions at the sub-population level [8, 13, 34, 39–44]. In West Africa, recommendations have been made to manage the small, isolated populations of lions as separate entities to allow for site-specific management and legislation [39–40]. The determination of regional sub-populations of lions in Zambia could be an important step for the creation of national wildlife management and legislation to preserve genetically healthy populations, ideally through the maintenance or restoration of natural connectivity at the landscape scale.

## Supporting Information

S1 Appendix Laboratory protocol. (DOCX)

S1 Table Sample information and haplotype calls for 165 lions. (XLSX)

S2 Table Polymorphic sites between all 12S-16S mitochondrial haplotypes. (DOCX)
