# Supplementary material for: Mitochondrial Haplotype Diversity in Zambian Lions: Bridging a Gap in the Biogeography of an Iconic Species
Source: PLoS One. 2015 Dec 16;10(12):e0143827. doi: 10.1371/journal.pone.0143827 (PMC4686026; doi:10.1371/journal.pone.0143827)
Supplement: S1 Appendix — (DOCX) [file pone.0143827.s001.docx]

S1 - Appendix

**Biopsy Tissue and Hide DNA Extraction:**

*Qiagen Kit DNA Isolation from Tissue Part 1*

1. Cut a small piece of tissue into a 1.7-mL tube for each sample
2. Add 300-μL Cell Lysis Solution to each tube
3. Add 5-μL Proteinase K to each tube
4. Vortex and incubate at 56⁰C overnight or until samples have lysed

*Qiagen Kit DNA Isolation from Tissue Part 2*

1. Place tubes on ice for 7min
2. Add 100-μL of protein precipitation solution
3. Vortex 20 sec (keep finger on the cap as they tend to open after ice) and spin for 3 min
4. Transfer supernatant to new tubes and precipitate DNA with an equal amount (≈300-μL) of 100% Isopropanol
5. Invert tubes 50x and spin for 3 min
6. Discard supernatant and wash DNA with 300-μL 70% Ethanol
7. Spin 2 min and discard supernatant
8. Allow to air dry and rehydrate DNA with 50-μL 1xTE

**Hair DNA Extraction:**

*KAPA Express Hair Extraction Protocol*

1. Rinse hair follicles with 70% ethanol and blot dry with kimwipe.
2. Cut 1 hair follicle into 0.2-mL tube.
3. Spin tubes briefly before starting extraction to ensure all follicles are at the bottoms of the tubes.
4. Thaw 10x Buffer, mix reagents as follow: (Mix 4 to 5 sample extra)
   - 10xBuffer 2-µL
   - Express Enyzme 0.2-µL
   - H_2_O 17.8-µL
   - Total 20-µL
5. Vortex buffer mixture & spin down briefly.
6. Transfer 20-uL mixture into 0.2-mL tubes containing hair follicles.
7. Vortex tubes & centrifuge briefly. Hair follicles MUST be in the solution before the incubation.
8. Place on thermal cycler at 75°C for 10 min, 95°C for 5 min.
9. Vortex tubes 5 sec & Spin at full speed (3500 rpm=4500g) in Hermle plate centrifuge for 2 min.
10. Transfer entire liquid portion to 0.65-mL Eppendorf tubes or into 96-well plates, leaving behind hair follicles. If needed, samples can be covered at this point and stored at 4°C overnight.
11. Using liquid from top portion of extraction, make a 1:10 dilution in 20-µL total volume for the all assays using purified WATER in new 0.65-mL tubes or 96-well plates (e.g. 2-µL DNA extract and 18-µL water). Frother 1:10 diluted DNA can be used for mtDNA assay.
12. Vortex and centrifuge dilutions briefly to pool liquid in bottom of tubes.
13. Store all samples at -20°C before use.
