# Supplementary material for: Mitochondrial Haplotype Diversity in Zambian Lions: Bridging a Gap in the Biogeography of an Iconic Species
Source: PLoS One. 2015 Dec 16;10(12):e0143827. doi: 10.1371/journal.pone.0143827 (PMC4686026; doi:10.1371/journal.pone.0143827)
Supplement: S2 Table — (DOCX) [file pone.0143827.s003.docx]

**S2 Table: Polymorphic sites between all 12S-16S mitochondrial haplotypes.** H1- H12 are haplotypes which were described by Antunes et al [13] and Z1-Z5 are novel haplotypes so far only found within Zambia.

| H1 | H2 | H3 | H4 | H5 | H6 | H7 | H8 | H9 | H10 | H11 | H12 | Z1 | Z2 | Z3 | Z4 | Z5 |  |
| --- | --- | --- | --- | --- | --- | --- | --- | --- | --- | --- | --- | --- | --- | --- | --- | --- | --- |
| H1 | 5 | 6 | 12 | 8 | 9 | 10 | 10 | 9 | 9 | 10 | 11 | 10 | 11 | 1 | 10 | 10 | H1 |
|  | H2 | 5 | 11 | 7 | 8 | 9 | 9 | 8 | 8 | 9 | 10 | 9 | 10 | 6 | 9 | 9 | H2 |
|  |  | H3 | 10 | 6 | 7 | 8 | 8 | 7 | 7 | 8 | 9 | 8 | 9 | 7 | 8 | 8 | H3 |
|  |  |  | H4 | 8 | 9 | 10 | 10 | 9 | 9 | 10 | 11 | 10 | 11 | 11 | 10 | 10 | H4 |
|  |  |  |  | H5 | 1 | 2 | 4 | 5 | 5 | 6 | 7 | 6 | 7 | 9 | 6 | 6 | H5 |
|  |  |  |  |  | H6 | 3 | 5 | 6 | 6 | 7 | 8 | 7 | 8 | 10 | 7 | 7 | H6 |
|  |  |  |  |  |  | H7 | 2 | 7 | 7 | 8 | 9 | 8 | 9 | 11 | 8 | 8 | H7 |
|  |  |  |  |  |  |  | H8 | 5 | 5 | 6 | 7 | 6 | 7 | 11 | 6 | 6 | H8 |
|  |  |  |  |  |  |  |  | H9 | 2 | 3 | 4 | 3 | 4 | 10 | 1 | 1 | H9 |
|  |  |  |  |  |  |  |  |  | H10 | 1 | 2 | 1 | 2 | 10 | 3 | 3 | H10 |
|  |  |  |  |  |  |  |  |  |  | H11 | 1 | 2 | 3 | 11 | 4 | 4 | H11 |
|  |  |  |  |  |  |  |  |  |  |  | H12 | 3 | 4 | 12 | 5 | 5 | H12 |
|  |  |  |  |  |  |  |  |  |  |  |  | Z1 | 1 | 11 | 4 | 4 | Z1 |
|  |  |  |  |  |  |  |  |  |  |  |  |  | Z2 | 12 | 5 | 5 | Z2 |
|  |  |  |  |  |  |  |  |  |  |  |  |  |  | Z3 | 11 | 11 | Z3 |
|  |  |  |  |  |  |  |  |  |  |  |  |  |  |  | Z4 | 2 | Z4 |
|  |  |  |  |  |  |  |  |  |  |  |  |  |  |  |  | Z5 | Z5 |
